# Supplementary material for: Linearly polarized GHz magnetization dynamics of spin helix modes in the ferrimagnetic insulator Cu2OSeO3
Source: Sci Rep. 2017 Aug 1;7:7037. doi: 10.1038/s41598-017-07020-2 (PMC5539291; doi:10.1038/s41598-017-07020-2)
Supplement: Supplementary file 1 — Supplementary Information [file 41598_2017_7020_MOESM1_ESM.pdf]

Supplementary Information on Linearly polarized GHz magnetization dynamics of spin helix modes in the ferrimagnetic insulator Cu_2OSeO_3

I. Stasinopoulos,[1] S. Weichselbaumer,[1] A. Bauer,[2] J. Waizner,[3] H. Berger,[4] M. Garst,[3,5] C. Pfleiderer,[2] and D. Grundler[6,*]

[1]*Physik Department E10, Technische Universität München, 85748 Garching, Germany*

[2]*Physik Department E51, Technische Universität München, 85748 Garching, Germany*

[3]*Institut für Theoretische Physik, Universität zu Köln, 50937 Köln, Germany*

[4]*Institut de Physique de la Matière Complexe, École Polytechnique Fédérale de Lausanne, 1015 Lausanne, Switzerland*

[5]*Institut für Theoretische Physik, Technische Universität Dresden, 01062 Dresden, Germany*

[6]*Institute of Materials and Laboratory of Nanoscale Magnetic Materials and Magnonics (LMGN), École Polytechnique Fédérale de Lausanne (EPFL), Station 12, 1015 Lausanne, Switzerland*

(Dated: June 6, 2017)

[*] Electronic mail: dirk.grundler@epfl.ch

I. EXPERIMENTAL ASPECTS

A. Crystals of Cu_2OSeO_3

Single crystals were grown by vapor transport [1]. Samples that crystallized in the space group $P2_13$ were oriented by Laue x-ray diffraction, cut with a wire saw, and carefully polished to size. The samples had a size of about $0.4 \times 2.3 \times 0.3\,\mathrm{mm}^3$. Their corresponding edges were collinear to crystallographic orientations along as follows: $\langle 110\rangle$, $\langle 110\rangle$ and $\langle 100\rangle$ (sample #1), $\langle 100\rangle$, $\langle 100\rangle$ and $\langle 100\rangle$ (sample #2), $\langle 110\rangle$, $\langle 211\rangle$ and $\langle 111\rangle$ (sample #3), respectively. In the main text we focus on sample #1. Samples #2 and #3 were used for additional measurements with a coplanar waveguide of small signal-line width.

B. Experimental setup

In Fig. 1a we show a photograph of sample #1 mounted in the cryostat. The coplanar

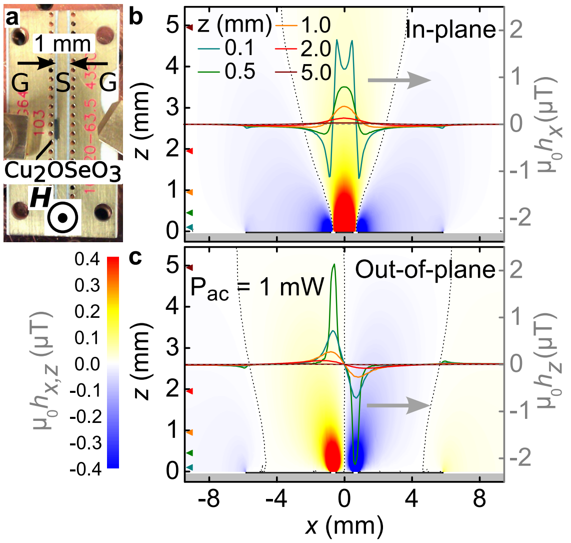

FIG. 1. **Experimental setup and waveguide far-field profile**. **a**, Sample #1 placed on top of the commercial back-grounded broad CPW used in this work. Here it is fixed in the gap between signal (S) and ground (G) line with a brass clamp. **b-c**, Far-field in- and out-of-plane amplitude profile of the dynamic magnetic field **h** for the broad CPW. Line plots show cuts at different heights above the CPW. An incident power of 1 mW and impedance of 50 Ω have been considered.

waveguide (CPW) was contacted via non-magnetic microwave probe tips (not shown; sup-

plier *GGB Industries*). Via holes provided electrical and thermal contact between ground lines and the copper sample stage, where the heater is mounted. The superconducting magnet of the *LakeShore* probe station CPX-VF provides a perpendicular field H of up to 2.5 T. Near- and far-field in- and out-of-plane amplitude profiles of the dynamic magnetic fields $\mathbf{h}$ have been calculated for different CPWs [2]. For the simulations we considered the layout of coplanar waveguides with different geometrical parameters such as widths of signal and ground lines, gaps and layer thicknesses (see Table I). This was done to optimize for

CPW	w_s (μm)	w_g (μm)	w_{sg} (μm)	t (μm)	metallization
broad	1000	5000	330	50	Cu
narrow	20	295	12.4	0.12	Cr/Au

TABLE I. **Dimensions of coplanar waveguides**. Signal line width w_s, ground line width w_g, gap width w_{sg}, and thickness t. The "broad CPW" is the commercial CPW used to obtain the data in this work.

an impedance of 50 Ω. Via holes in the commercial CPW contacted an additional metallic ground plane on the backside of the CPW substrate [Fig. 1a]. They redistributed the back-flowing radiofrequency currents in the ground lines, but were not expected to alter considerably the strong magnetic field components close to the signal line [3]. Hence, they were not considered in the simulations. Field profiles of the broad CPW are summarized in Fig. 1b,c for a larger parameter space compared to Fig. 1b of the main text. At the edges of the metallic leads, the field amplitudes were enhanced due to the finite penetration depth of the rf current (skin effect).

C. Coplanar waveguide of small signal-line width

We used also a narrow CPW (signal line width of 20 μm) on which a bar-shaped crystal of Cu_2OSeO_3 (sample #2) was positioned along the signal line and covered both the signal line and the gaps ($N_x \gg N_y$). In agreement with the considerations presented in the manuscript, the spectra (not shown) contained three modes in the SkL phase and a single resonance in the conical state attributed to the $+Q$ mode. We placed a further sample (sample #3) in the same way on the narrow CPW as sample #2. Here, the high symmetry direction $\langle 111 \rangle$ was collinear with the applied field H. The spectra resembled the ones observed for

H parallel $\langle 100 \rangle$. We encountered a small shift of eigenfrequencies only, consistent with the cubic anisotropy being the weakest energy scale in the chiral magnet [4].

D. Sample temperature

In our experiment the sample temperature was measured at the sample stage, which is thermally anchored to a cold finger. For each probing configuration described in this work, there was a slightly different thermal contact between the sample, the CPW and the sample stage. At each cool down, we chose a temperature T in the vicinity of 57 K, where the SkL was stabilized. The exact values for T and the H_{c2} values used to normalize the data in Fig. 2e-g of the main text are given in Tab. II.

Probing configuration	**a**	**b**	**c**	**d**
nominal T (K)	57.3	57.5	56.5	56.5
$\mu_0 H_{c2}$ (mT)	45	48	58	58

TABLE II. **Temperature at the sample stage & critical field values**. Nominal temperature and extracted values of the critical field H_{c2} for the different probing configurations shown in Fig. 2a-d. The uncertainty in $\mu_0 H_{c2}$ is estimated to be ± 1 mT.

E. Detailed set of spectra

We recorded the scattering parameter $|S_{12}|(f)$ by varying the frequency f. To enhance the signal-to-noise ratio, we made use of the difference technique in that we analyzed $\Delta|S_{12}| = |S_{12}| - |S_{12}^{\text{ref}}|$. S_{12}^{ref} was taken at 1 T and did not contain the resonances of interest. In Fig. 2, we show the detailed datasets from which we extracted the field dependencies of eigenfrequencies.

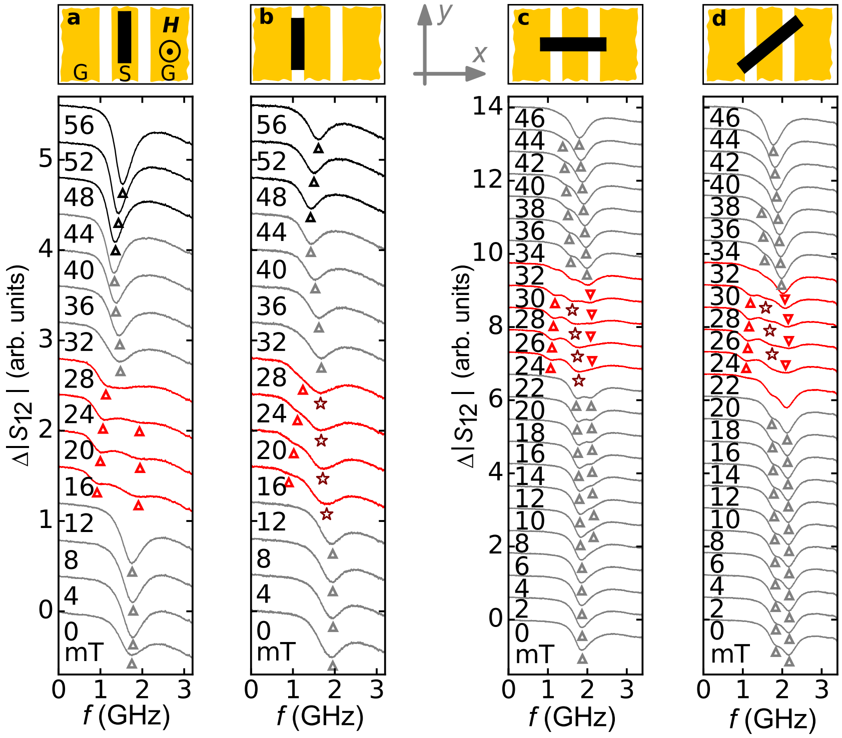

FIG. 2. **Detailed set of spectra**. Data used to extract the field dependencies of resonance frequencies of the sample when placed differently on the broad coplanar waveguide. Spectra taken in the field-polarized (FP) phase are shown in black. Spectra are recorded for successively decreased fields and are shifted on the vertical axis for clarity. Symbols are introduced to mark resonance frequencies (compare Fig. 2 of the main text). Some spectra are taken at field values that coincide with a phase boundary, where phases might have coexisted in the macroscopic sample due to inhomogeneities. Here, the mode allocation is not clear and omitted.

II. POLARIZATION AND ELLIPTICITY OF MAGNETIZATION PRECESSION

A. Ferromagnets

According to the orthodox understanding of magnetic resonance [5], the ellipticity ε of a general ellipsoid of a field-polarized ferromagnetic material is given by

$$\varepsilon = \sqrt{(N_y - N_x)/(N_y - N_z + H/M)}, \tag{1}$$

where directions $\hat{x}, \hat{y}, \hat{z}$ and magnetic field $\mathbf{H}$ are oriented as sketched in Fig. 1a of the main text. M is the magnetization and N_i $(i = x, y, z)$ are the components of the demagnetization tensor. For Eq. (1), one assumes $N_y \geq N_x$ and $N_x + N_y + N_z = 1$ with the external field H

applied along the ellipsoidal $\hat{z}$ axis [5].

For a cylindrical sample shape $N_y = N_x$ the oscillating magnetization is circularly polarized with ellipticity $\varepsilon = 0$. For $N_y > N_x$ the polarization is elliptically deformed so that $\varepsilon > 0$. The maximal value of ε is obtained in the limit of zero internal field $H_{\rm int} = H - N_z M$ where

$$\varepsilon(H_{\rm int} = 0) = \sqrt{1 - N_x/N_y}, \tag{2}$$

for which the Kittel resonance frequency reduces to

$$\omega(H_{\rm int} = 0) = \gamma\mu_0 M\sqrt{N_x N_y}. \tag{3}$$

In order to obtain $\epsilon \to 1$, we get $N_x/N_y \to 0$. At the same time, however, according to Eq. (3) the resonance frequency vanishes. A linear polarization of the uniform mode in a ferromagnet in not anticipated. The linear polarization in chiral magnets reported in our manuscript is very robust and arises for any generic sample shape, as it is controlled by the symmetries of the helical spin structure that is stabilized by the Dzyaloshinskii-Moriya-interaction.

B. Chiral magnets

The field dependence of the eigenfrequencies in Cu_2OSeO_3 as well as ellipticities were modelled in the framework of the theory outlined in Ref. [6] by T. Schwarze *et al.*. The result depends on the internal conical susceptibility $\chi_{\rm con}^{\rm int}$, i.e., the magnetic susceptibility within the conical phase with $\chi_{\rm con}^{\rm int} = 1.76$ for Cu_2OSeO_3, and the temperature dependent critical field $H_{c2}(T)$ (see Table II) where the transition into the field-polarized phase occurs. The sample shape is modelled by an ellipsoid with demagnetization factors N_x, N_y, and N_z where the field is applied along the principal $\hat{z}$ axis.

1. Ellipticity for the helix at zero and finite magnetic fields

The mean magnetization for the $+Q$ and $-Q$ modes of the helix, $\sigma = 1$ and $\sigma = -1$, respectively, oscillates, and its dynamic part has the form

$$\mathbf{m}_\sigma(t) = \begin{pmatrix} m_\sigma^x \cos(\omega^\sigma t) \\ \sigma m_\sigma^y \sin(\omega^\sigma t) \\ 0 \end{pmatrix} \tag{4}$$

where we assumed that the pitch is aligned with the principal $\hat{z}$ axis, and m_σ^x and m_σ^y are the amplitudes along the principal $\hat{x}$ and $\hat{y}$ axes. An explicit expression for the eigenfrequencies ω^σ was given in Ref. [6]. The mean magnetization oscillates counterclockwise for the $+Q$ mode and clockwise for the $-Q$ mode within the plane perpendicular to the pitch, i.e., the $\hat{z}$ axis. We neglected the influence of cubic anisotropies which will be discussed elsewhere. Neglecting these cubic anisotropies, we obtain for the ratio of amplitudes

$$\frac{m_\sigma^x}{m_\sigma^y} = \left| \frac{2h^2 N_x N_y \chi_{\text{con}}^{\text{int}} + \sigma W_1 - (N_x - N_y)(2 + (1-h^2)\chi_{\text{con}}^{\text{int}}) - 2N_x h W_{\sigma,2}}{2h^2 N_x N_y \chi_{\text{con}}^{\text{int}} + \sigma W_1 + (N_x - N_y)(2 + (1-h^2)\chi_{\text{con}}^{\text{int}}) - 2N_y h W_{\sigma,2}} \right| \tag{5}$$

where we abbreviated $W_1, W_{\sigma,2} > 0$ with

$$\begin{aligned} W_1^2 = {} & (N_x - N_y)^2 (2 + (1-h^2)\chi_{\text{con}}^{\text{int}})^2 + \\ & 4h^2 N_x N_y ((2 + \chi_{\text{con}}^{\text{int}})(4 + \chi_{\text{con}}^{\text{int}}(N_x + N_y)) - h^2(4 + \chi_{\text{con}}^{\text{int}}(4 + \chi_{\text{con}}^{\text{int}}(N_x + N_y) - \chi_{\text{con}}^{\text{int}} N_x N_y))) \end{aligned} \tag{6}$$

$$\begin{aligned} W_{\sigma,2}^2 = {} & \sigma W_1 \chi_{\text{con}}^{\text{int}} + (2 + \chi_{\text{con}}^{\text{int}})(4 + \chi_{\text{con}}^{\text{int}}(N_x + N_y)) \\ & - h^2(4 + \chi_{\text{con}}^{\text{int}}(4 + \chi_{\text{con}}^{\text{int}}(N_x + N_y) - 2\chi_{\text{con}}^{\text{int}} N_x N_y)). \end{aligned} \tag{7}$$

The result depends on the constant internal susceptibility of the conical phase $\chi_{\text{con}}^{\text{int}} = \chi_{\text{con}}/(1 - \chi_{\text{con}} N_z)$, the demagnetization factors N_x, N_y, N_z, and the magnetic field $h = H/H_{c2}$ where H_{c2} is the critical field. The ellipticity $\varepsilon_\sigma > 0$ is given by

$$\varepsilon_\sigma = \frac{\sqrt{|(m_\sigma^x)^2 - (m_\sigma^y)^2|}}{\max(m_\sigma^x, m_\sigma^y)}. \tag{8}$$

In the main text, we also introduced a negative ellipticity in case $m_\sigma^y > m_\sigma^x$ and positive otherwise.

In the limit $h \to 0$, the ellipticity reduces to a step function as discussed in the main text. In the other limit $h \to 1^-$, the ellipticity of the $+Q$ mode, ε_+, reduces to the expression of Eq. (1) for a field-polarized magnet. The $+Q$ mode has the same handedness as the Kittel

mode so that they are smoothly transformed into each other at the second-order transition at the critical field H_{c2}.

Interestingly, the $+Q$ mode changes the polarization axes at some intermediate field $h_+^{\rm circ}$ as can be seen in Fig. 3b of the main text. The $+Q$ mode is circular polarized, $\varepsilon_+ = 0$, at this field $h_+^{\rm circ}$ irrespective of demagnetization factors N_x and N_y. We obtain for this field the explicit expression

$$h_+^{\rm circ} = \frac{2+\chi_{\rm con}^{\rm int}}{\sqrt{(2+\chi_{\rm con}^{\rm int})(2+(2-N_z)\chi_{\rm con}^{\rm int}) + \sqrt{(1-N_z)\chi_{\rm con}^{\rm int}(2+\chi_{\rm con}^{\rm int})^2(4+(1-N_z)\chi_{\rm con}^{\rm int})}}}. \quad (9)$$

For our Cu_2OSeO_3 sample with $\chi_{\rm con}^{\rm int} = 1.76$ and $N_z = 0.53$ we get $h_+^{\rm circ} \approx 0.76$.

2. Ellipticity in the Skyrmion lattice phase

In Fig. 3 we show the results of our calculation for the ellipticity and spectral weight distribution of the gyrational Skyrmion lattice (SkL) modes, CCW and CW. We concentrate on field values around 0.5 $\mathrm{H_{c2}}$, where the SkL phase is stable. We find that linear polarization, i.e. $|\varepsilon| = 1$ is not achieved for any sample shape. The spectral weight Γ of the counter-clockwise (CCW) mode is larger compared to the clockwise (CW) mode for every sample shape. The mean magnetization oscillates counter-clockwise and clockwise for the CCW and CW mode, respectively, within the plane perpendicular to the applied magnetic field. The spectral weight of the CCW mode increases with increasing field H. For the CW mode one finds the opposite trend. Note that the ellipticity of the CCW mode is practically independent of the field and already well described by the Kittel expression of Eq. (1) indicating that it is basically dominated by the polarized background. As the CCW and CW modes are elliptically polarized, either sample placement that covers CPW signal line *and* gaps allows one to monitor them in the same spectrum. The breathing mode is linearly polarized by symmetry and can be excited only with the help of a longitudinal dynamical field h_z.

---

[1] Gnezdilov, V. P. *et al.* Magnetoelectricity in the ferrimagnetic Cu_2OSeO_3: symmetry analysis and Raman scattering study. *Low Temp. Phys.* **36**, 550–557 (2010). URL `http://scitation.aip.org/content/aip/journal/ltp/36/6/10.1063/1.3455808`.

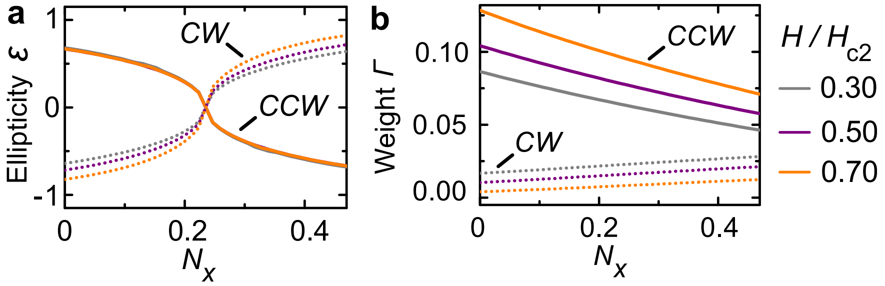

FIG. 3. Ellipticity and spectral weight in the SkL phase. **a**, Ellipticity ε of the gyrational SkL modes as a function of the demagnetization factor N_x ($N_z = 0.53$) calculated for different magnetic fields (colors), at which the SkL is stable. Solid and dotted lines represent the counter-clockwise (CCW) and clockwise (CW) SkL modes, respectively. **b**, Spectral weight Γ of SkL modes for an excitation field $\mathbf{h}$ along $\hat{x}$.

[2] The software *CST Microwave Studio 2014* (CST Computer Simulation Technology, www.cst.com) has been used for optimizing the impedance to 50 Ω and visualize the field profile of the CPW.

[3] Simons, R. N. *Coplanar Waveguide Circuits, Components, and Systems* (John Wiley and Sons Inc., 2001).

[4] Bak, P. & Jensen, M. H. Theory of helical magnetic structures and phase transitions in MnSi and FeGe. *J. Phys. Condens. Matter* **13**, L881 (1980). URL `http://stacks.iop.org/0022-3719/13/i=31/a=002`.

[5] Gurevich, A. G. & Melkov, G. A. *Magnetization Oscillations and Waves* (CRC Press, Boca Raton, 1996).

[6] Schwarze, T. *et al.* Universal helimagnon and skyrmion excitations in metallic, semiconducting and insulating chiral magnets. *Nature Mater.* **14**, 478–483 (2015). URL `http://dx.doi.org/10.1038/nmat4223`.
